# The mitochondrial genome of a 9-arm feather star *Thaumatocrinus naresi* (Crinoidea, Pentametrocrinidae)

**DOI:** 10.1080/23802359.2023.2184651

**Published:** 2023-03-08

**Authors:** Qinyi Lin, Ruiyan Zhang, Chunsheng Wang

**Affiliations:** a School of Oceanography, Shanghai Jiao Tong University, Shanghai, China; b Key Laboratory of Marine Ecosystem Dynamics, Second Institute of Oceanography, Ministry of Natural Resources, Hangzhou, China; c Southern Marine Science and Engineering Guangdong Laboratory (Zhuhai), Zhuhai, China

**Keywords:** Western Pacific, mitogenome, phylogeny, crinoid, Comatulida, *Thaumatocrinus naresi*

## Abstract

The genus *Thaumatocrinus* is composed of species displaying 10 undivided arms arising from 10 radials. *Thaumatocrinus naresi* is also considered to be a 10-arm species, but individuals with 9 arms were also reported. Here, we report the mitochondrial genome of *T. naresi* with 9 arms collected from the western Pacific. The genome is 16,047 bp in length with a 67.84% AT content. It contains 13 protein-coding genes (PCGs), 2 ribosomal RNA genes, and 22 transfer RNA genes. Phylogenetic analysis shows that *T. naresi* forms an independent lineage within Comatulida.

## Introduction

*Thaumatocrinus* Carpenter, 1883 is a genus feather star in the family Pentametrocrinidae A. H. Clark 1908, composed of 6 species with 10 arms (Carpenter 1883, 1884, 1888; Clark 1907, 1908, 1915; Clark and Clark 1967). Individuals with fewer arms in the genus *Thaumatocrinus* were also frequently spotted (Clark 1908, 1915). In addition, juvenile specimens of *Thaumatocrinus* were reported to have only 5 arms and radials (Carpenter 1884; Clark 1915). In this study, the complete mitochondrial genome from a 9-arm *T. naresi* is described, and a phylogenetic tree of *T. naresi* and other crinoid species is included.

## Materials and methods

**Figure 1.**
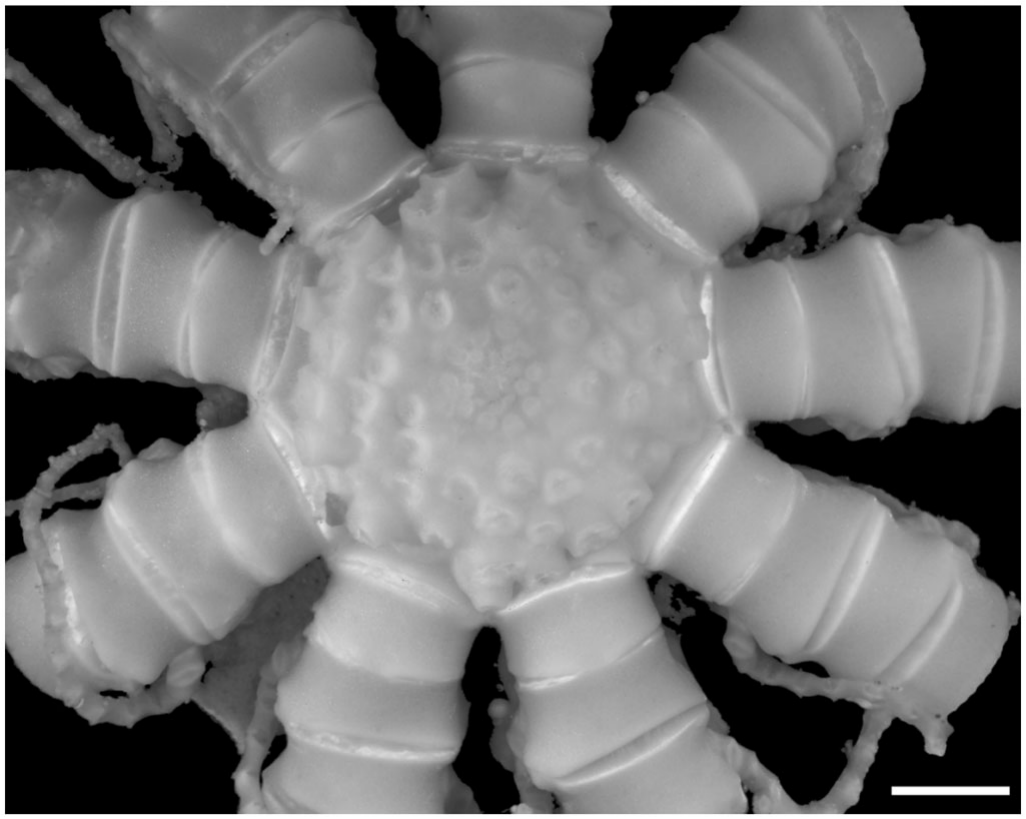
RSIOCR118 deposited in the sample repository of SIO, MNR, Hangzhou, China, scale bar 2 mm, taken by Qinyi Lin.

The specimen was collected alive from the western Pacific (162°49′43″E, 15°10′46″N, 1318 m depth) and identified as *Thaumatocrinus naresi* based on its morphological characters (Figure 1).

The specimen (RSIOCR118) and its DNA are deposited in the Sample Repository of the Second Institute of Oceanography, Ministry of Natural Resources, Hangzhou, China (Chunsheng Wang, wangsio@sio.org.cn). DNA was extracted with QIAamp Tissue Kit (QIAGEN, Hilden, Germany) and mitochondrial DNA was amplified with a DNA REPLI-g Mitochondrial DNA Kit (QIAGEN, Hilden, Germany). Library construction and sequencing were performed by Biozeron (Biozeron, Shanghai, China) using the Illumina novaseq 6000 sequencing platform (Illumina, San Diego, CA, USA). The raw data was assembled using GetOrganelle v1.7.5 (Freudenthal et al. 2020), and genome annotations were performed using MITOS (Bernt et al. 2013). The phylogenetic analysis was conducted by the maximum likelihood (ML) method using MEGA 11 (Tamura-Nei model) with 1000 replication of bootstrap assembling (Tamura et al. 2021).

## Results

**Figure 2.**
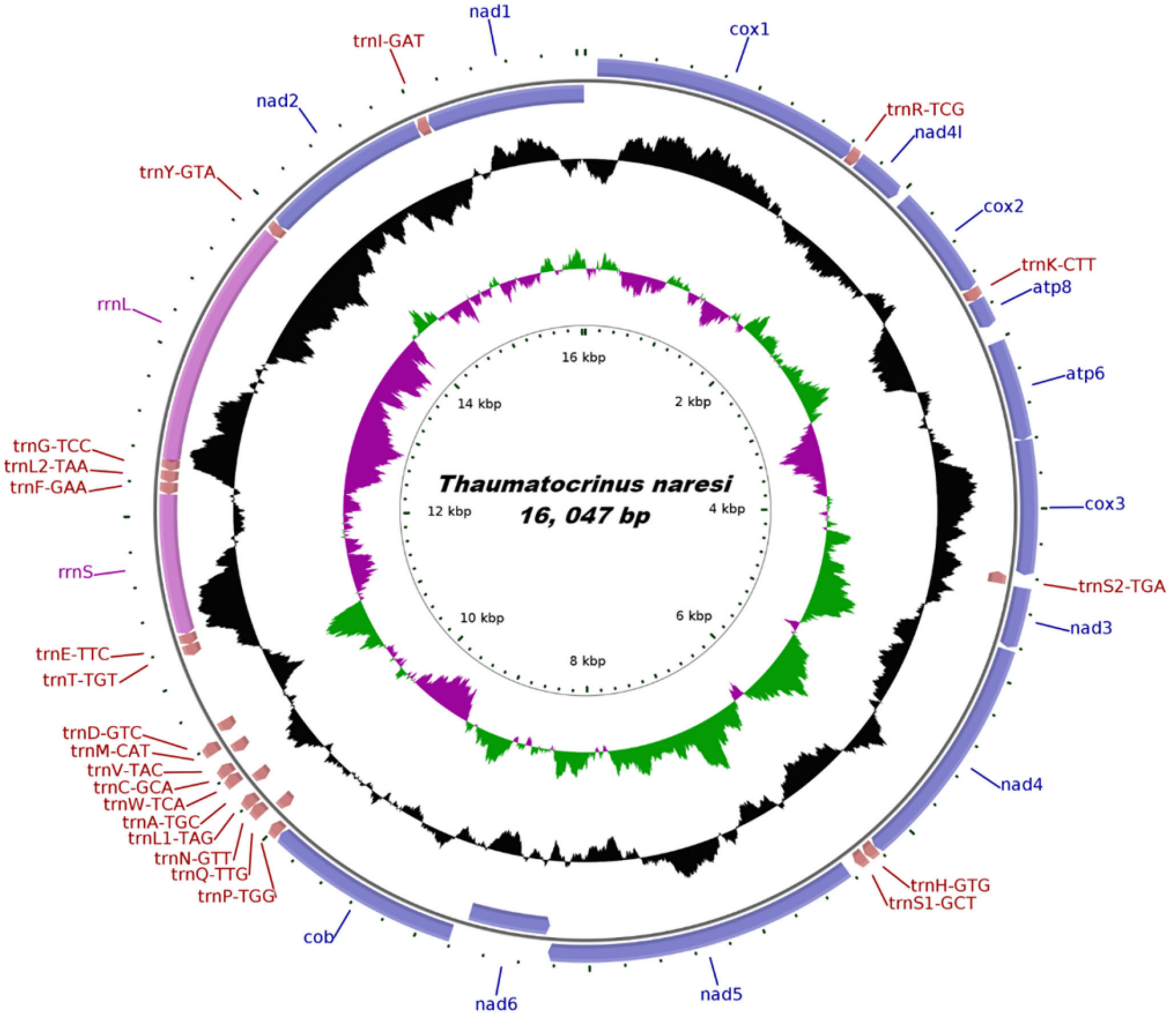
Mitochondrial gene map of *Thaumatocrinus naresi.*

The complete mitogenome sequence of *T. naresi* is 16,047 bp in length with a 67.84% AT content. It contains 13 protein-coding genes (PCGs), 2 ribosomal RNA genes, and 22 transfer RNA genes (Figure 2). Among the 37 genes, both rRNA genes (rrnL and rrnS) are encoded on the light strand. Twelve tRNA genes (*trnS2-tga, trnQ-ttg trnA-tgc, trnV-tac, trnD-gtc, trnT-tgt, trnE-ttc, trnF-gaa, trnL2-taa, trnG-tcc, trnY-gta* and *trnI-gat*) are encoded on the light strand. Only three PCGs (nad1, nad2, and nad6) are encoded on the light strand, whereas the other ten PCGs are located on the heavy strand. Twelve PCGs (*cox1, cox2, cox3, cob, nad1, nad2, nad3, nad4, nad4L, nad6, atp8*, and *atp6*) are initiated by ATG. One PCG (nad5) is started by GTG. Six PCGs (*cox1, cox3, atp6, nad1, nad2*, and *nad4L*) terminate with the typical TAA as stop codon, while seven PCGs (*cox2, cob, atp8, nad3, nad4, nad5*, and *nad6*) end with TAG. The 22 transfer RNA genes range in size from 68 to 73 bp. The gene arrangement of *T. naresi* was in accord with other crinoids, except *Antedon mediterranea* (AM404181), *Neogymnocrinus richeri* (DQ068951). The mitogenome of *T. naresi* is deposited in GenBank, under accession number OP428702.

## Discussion and conclusion

**Figure 3.**
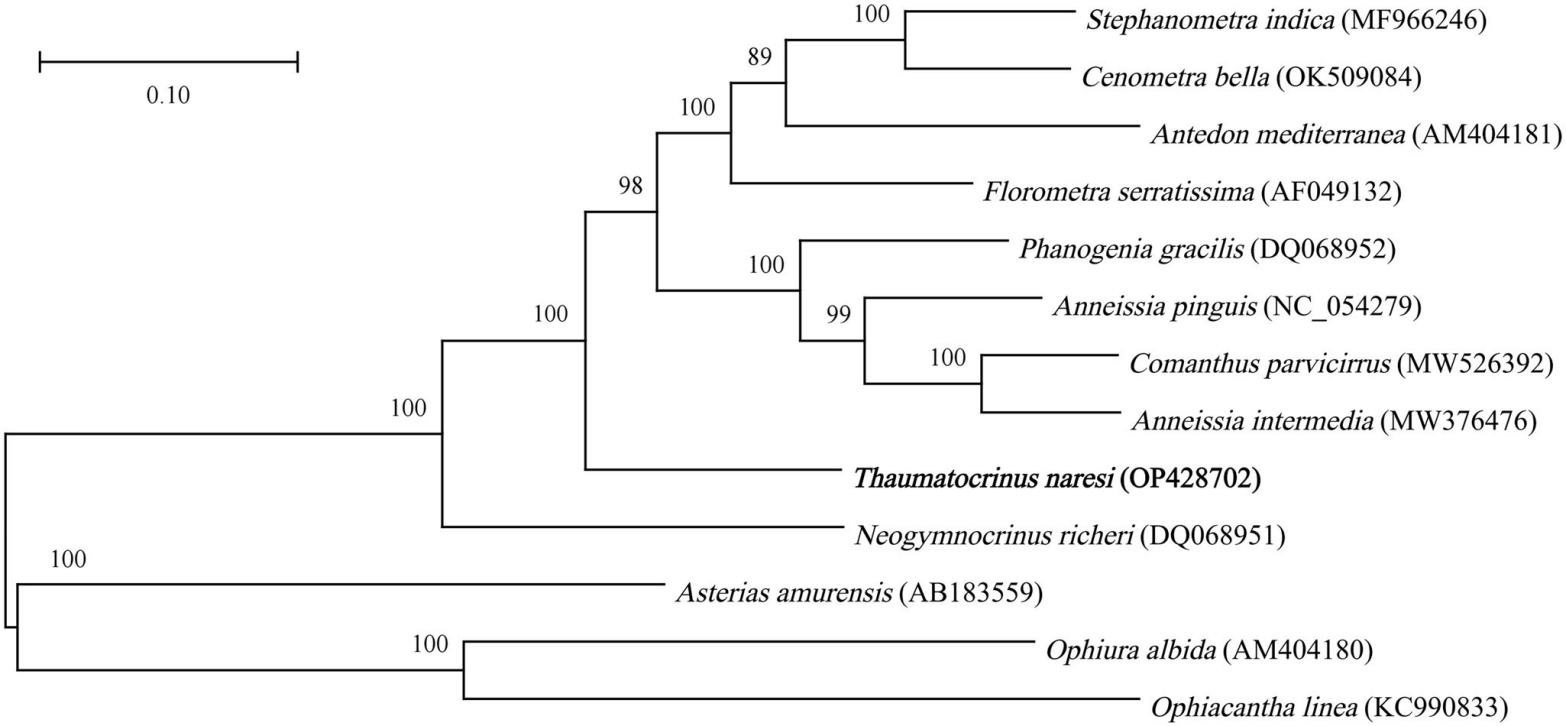
Phylogenetic tree of *Thaumatocrinus naresi* and other mitogenomes from Crinoidea, Ophiuroidea, and Asteroidea based on mitochondrial PCGs.

The phylogenetic relationship of *T. naresi* with other crinoids was analyzed. 12 mitochondrial genomes were obtained from the GenBank database (9 from Crinoidea, 2 from Ophiuroidea, and 1 from Asteroidea). The tree topologies showed that *T. naresi* fell within the cluster comprising Comatulida species and formed an independent lineage (Figure 2) with a high bootstrap value (100). In this research, the complete mitochondrial genome sequence of *T. naresi* was provided. Given that abundant mitochondrial genomic data and comprehensive analysis are still required to discover the phylogeny and evolution of crinoids, this study is an important contribution to this field (Figure 3).

## Ethical approval

Study species were crinoids collected in International Waters and permission for sampling was not necessary. Animals were preserved in ethanol.

## Data Availability

The genome sequence data that support the findings of this study are openly available in GenBank of NCBI at [https://www.ncbi.nlm.nih.gov] under the accession no. OP428702. BioProject, SRA, and Bio-Sample numbers are PRJNA880019, SRR21615206, and SAMN30825162, respectively.
